# Rat bite fever due to *Streptobacillus notomytis* complicated by meningitis and spondylodiscitis: a case report

**DOI:** 10.1186/s12879-021-06715-2

**Published:** 2021-09-28

**Authors:** Suchada Pongsuttiyakorn, Witchuda Kamolvit, Sunee Limsrivanichakorn, Arissa Phothisirisakulwong, Walaiporn Wangchinda

**Affiliations:** 1 Division of Infectious Diseases and Tropical Medicine, Department of Medicine, Faculty of Medicine Siriraj Hospital, Mahidol University, 2 Wanglang Road, Bangkoknoi, Bangkok, 10700 Thailand; 2 Department of Microbiology, Faculty of Medicine Siriraj Hospital, Mahidol University, Bangkok, Thailand; 3 Division of Diagnostic Radiology, Department of Radiology, Faculty of Medicine Siriraj Hospital, Mahidol University, Bangkok, Thailand

**Keywords:** Rat bite fever, *Steptobacillus*, *Streptobacillus notomytis*, Meningitis, Spondylodiscitis

## Abstract

**Background:**

Only three other cases of rat bite fever caused by *Streptobacillus notomytis* in humans have been reported since this species was identified in 2015. Data specific to the differences in clinical features and geographic distribution between *S. notomytis* infection and *S. moniliformis* infection are scarce. All previous cases of human *S. notomytis* infection were reported from Japan. This is the first case of *S. notomytis* infection reported from outside of Japan.

**Case presentation:**

A 72-year-old Thai woman was admitted to Siriraj Hospital (Bangkok, Thailand)—Thailand’s largest university-based national tertiary referral center—in August 2020 with fever, myalgia, and polyarthralgia for 3 days, and gradually decreased consciousness for the past 1 day. Physical examination and laboratory investigations revealed septic arthritis of both knee joints, meningitis, and hepatitis. She was initially misdiagnosed as rheumatoid arthritis in the elderly since the initial investigations were unable to detect a causative pathogen. However, *S. notomytis* infection was later confirmed by polymerase chain reaction amplification of a part of the 16S rRNA gene and sequencing from synovial fluid. Her clinical course was also complicated by spondylodiscitis and epidural abscess caused by *S. notomytis*, which was detected from tissue biopsy. Therefore, rat bite fever in this patient manifested as meningitis, septic polyarthritis, hepatitis, and spondylodiscitis. The patient was treated with intravenous ceftriaxone then switched to oral amoxicillin with complete recovery.

**Conclusions:**

The clinical manifestations of *S. notomytis* infection are similar to those demonstrated in *S. moniliformis* infection. This case also showed that arthritis caused by *S. notomytis* mimics rheumatoid arthritis, and that meningitis and spondylodiscitis are potential coexisting complications that can be found in *S. notomytis* infection.

## Background

*Streptobacillus* is a genus of fastidious Gram-negative bacteria having forms that include filaments, chains, and curved rods. *Streptobacillus moniliformis* is predominantly found in the upper respiratory tract of rat, and is known as the main pathogenic cause of rat bite fever [1]. Since 2014, four novel species named *Streptobacillus canis*, *Streptobacillus notomytis*, *Streptobacillus felis*, and *Streptobacillus ratti* have been described according to the genomic studies of *Streptobacillus* [2, 3]. Herein, we present a case of *S. notomytis* infection causing polyarthritis, meningitis, and spondylodiscitis. To the best of our knowledge, this is the first case report of human *S. notomytis* infection to be reported outside of Japan.

## Case presentation

A 72-year-old Thai woman was admitted to Siriraj Hospital (Bangkok, Thailand) in August 2020 with gradually decreasing consciousness over the preceding 1 day. During the preceding 3 days, she had fever, myalgia, pain, and swelling involving both knees and all joints of both hands. Her medical history was unremarkable except for cervical degenerative disc disease with spine surgery 15 years earlier.

The patient was febrile and drowsy on admission, and physical examination revealed neck stiffness without focal neurological deficits. Arthritis was noted at both knees, both wrists, and all joints of both hands. She had no rashes, and other examinations were unremarkable.

Laboratory investigation is shown in Table 1. Computed tomography of brain was normal. Lumbar puncture was performed and the CSF results are demonstrated in Table 1. Synovial fluid of both knees drawn by arthrocentesis was slightly turbid with a nucleated cell count of 12,900/uL with 94% neutrophils, and nucleated cell count of 69,300/uL with 95% neutrophils from the left knee and right knee, respectively. The patient was initially diagnosed with acute bacterial meningitis and polyarticular bacterial septic arthritis. After taking blood culture, high-dose intravenous ceftriaxone was administered for empirical treatment.

**Table 1:** Laboratory blood and CSF-data

Laboratory test	Patient results	Normal values
Blood		
White blood cell count	18,900/µL	4400–10,300/µL
Neutrophil	86%	40–73%
C-reactive protein	211.6 mg/L	< 5 mg/L
Alanine aminotransferase	60 IU/L	0–33 IU/L
Alkaline phosphatase	669 IU/L	35–104 IU/L
Creatinine	2.49 mg/dL	0.51–0.95 mg/dL
Cerebrospinal fluid		
Nucleated cell count	10/µL	
Neutrophils	75%	
Protein	89 mg/dL	15–45 mg/dL
Glucose	58 mg/dL	40–70 mg/dL
CSF-to-blood glucose ratio	0.36	

After 1 week of ceftriaxone treatment, the patient started to regain consciousness, but her fever and polyarthritis were not improved. The aerobic and anerobic cultures of blood, CSF, initial synovial fluid, and four-repeated synovial fluid were all negative. FilmArray^®^ Meningitis/Encephalitis Panel test (The BioFire^®^, BioMérieux) of CSF was negative. PCR amplification of a part of the 16S rRNA gene followed by sequencing of initial synovial fluid was unable to detect bacterial DNA. However, PCR amplification of 16S rRNA of CSF was not performed. Since autoimmune disease was a differential diagnosis, a rheumatologist was consulted. Dexamethasone was prescribed for treatment of suspected rheumatoid arthritis in the elderly. PCR amplification of a part of the 16S rRNA gene and sequencing of repeated synovial fluid from right knee was performed allowing the detection and identification of *Streptobacillus notomytis.* Singleplex PCR assay was undertaken in a final volume of 50 μL using Taq PCR Master Mix (Qiagen) with a final concentration of 0.2 μM for each primer. The two pairs of primers used in the assay were 16S-F1 5-AGAGTTTGATCMTGGCTCAG-3ʹ, 16S-R5 5ʹ-GCGTGGACTACCAGGGTATC-3ʹ and 16S-F3 5ʹ-CGGCTAACTCCGTGCCAGCA-3ʹ, 16S-R1 5ʹ-ACGGYTACCTTGTTACGACT-3ʹ. The amplification conditions were 94 °C for 5 min, followed by 30 cycles of 94 °C for 30 s, 56 °C for 45 s and 72 °C for 1 min 30 s, and final extension for 7 min. PCR products were sequenced in both directions by Sanger sequencing and submitted to GenBank under Accession number MZ676036. Rat bite fever due to *S. notomytis* was diagnosed and dexamethasone was discontinued. From retrospective history-taking, the patient denied any recent travel or animal bite, but she stated that there was group of rats living in her living area.

**Fig. 1:**
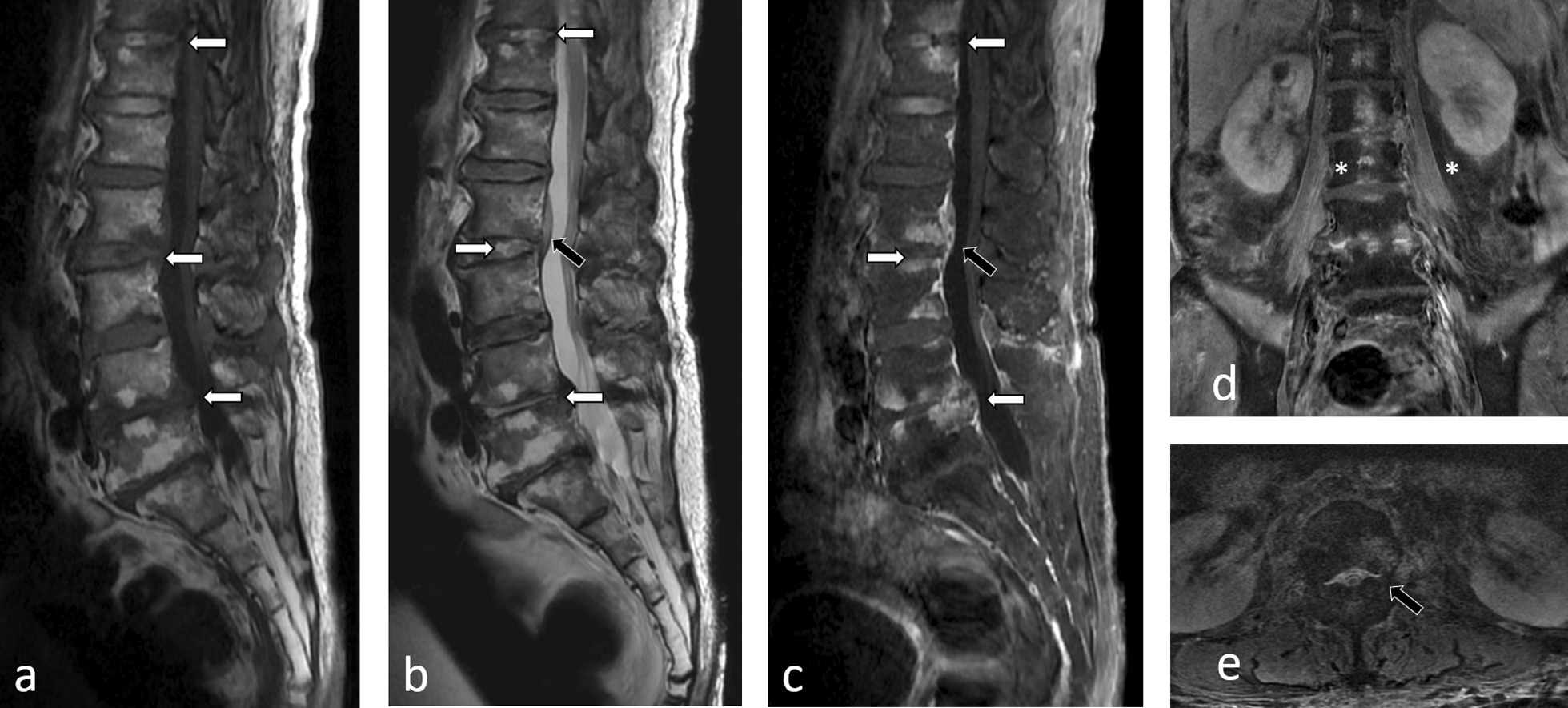
Magnetic resonance (MR) images of the lumbosacral spine. **a** Sagittal T1-weighted MR image shows endplate irregularity and abnormal low signal intensity (SI) in bone marrow at the T11–T12, L2–L3 and L4–L5 levels (white arrow). **b** Sagittal T2-weighted MR image shows high SI in the intervertebral disc of T11–T12, L2–L3 and L4–L5 levels (white arrow) and an epidural abscess (black arrow). **c** Sagittal contrast-enhanced (CE) fat-suppressed T1-weighted MR image shows enhancement of the endplates and bone marrow at the T11–T12, L2–L3 and L4–L5 levels (white arrow) with epidural abscess at L2–L3 level (black arrow). **d** Coronal CE fat-suppressed T1-weighted MR image shows faint enhancement at bilateral psoas muscles (asterisk) without abscess, represented myositis. **e** Axial CE fat-suppressed T1-weighted MR image shows an epidural abscess (black arrow)

During the second week of hospitalization, the patient complained of low back pain. Magnetic resonance imaging of the LS spine revealed findings compatible with T11–T12, L2–L3, and L4–L5 spondylodiscitis and epidural abscess (Fig. 1). Open debridement and biopsy were then performed. The pathologic findings showed acute suppurative inflammation. Tissue culture for bacteria, mycobacteria and fungi showed negative results; however, PCR amplification and sequencing detected *S. notomytis*. Therefore, rat bite fever in this patient manifested as meningitis, septic polyarthritis, and spondylodiscitis. The diagnosis of meningitis in this patient was made by only clinical presentations of alteration of consciousness with meningeal irritation sign; however, this could be overinterpreted since the CSF findings that showed mild elevation of cell count and decrease of glucose ratio could be from parameningeal inflammation from spondylodiscitis and epidural abscess. Abnormal liver tests at presentation were also then suspected as reflecting hepatitis due to rat bite fever.

The patient was treated with intravenous ceftriaxone for 35 days with partial recovery with a subsequent switch to oral amoxicillin 2 g/day for an additional 12 weeks. Arthritis affecting both hands and knees improved, C-reactive protein and liver enzymes decreased to within normal range, and the patient completely recovered.

## Discussion and conclusions

*Streptobacillus notomytis* is a novel species in the *Streptobacillus* genus that can cause rat bite fever; however, human infection by this species is rare. Only three cases have been reported since its discovery, and all three cases were reported from Japan [4–6]. The case reported herein is the first human case of *S. notomytis* infection reported from outside of Japan, and the first case reported from Thailand. The disease distribution appears to predominate in Asia, which is similar to rat bite fever caused by *Spirillum minus* [7]. In contrast, the disease distribution of rat bite fever caused by *S. moniliformis* infection does not restrict in any specific geographic area [8]. This finding could be explained by the hypothesis that the black rats (*Rattus rattus*) which are reported to be naturally colonized with *S. notomytis* commonly confined to warmer areas such as Asia and Africa, whereas the Norway rats (*Rattus norvegicus*) which are commonly colonized with *S. moniliformis* tend to be found in cooler regions and urban areas [4, 9]. *S. notomytis* and *S. moniliformis* infection in humans are believed to be underdiagnosed because it is a fastidious bacterium that requires special conditions for culture, and because PCR, which plays an important role in the diagnosis of *S. notomytis*, is not available in some centers. *S. notomytis* was first isolated in the Asia–pacific region in a spinifex hopping mouse in Australia, and in black rats in Japan [10]. However, *S. notomytis* was also isolated from house rats in a German zoo [11]. Therefore, the true incidence and area distribution of this microorganism is not yet known.

*Streptobacillus moniliformis* is transmitted to humans via the bite, scratch, or indirect contact of rats, and also via ingestion of food or water contaminated with rat excrement. The epidemiological link between rat exposure and human *S. notomytis* infection was confirmed in previous study [6]. No finding or evidence of *S. notomytis* harboring in any other types of animals has been published. Even though the exact cause of infection in our case remains unclear, the most likely cause is the group of rats that the patient had reported living in her living area.

Rat bite fever caused by *S. moniliformis* is typically characterized by fever, arthralgia and skin rash. Other complications, such as meningitis, endocarditis, hepatitis, and/or focal abscess, can be present in some patients [12]. Mortality in untreated patients is approximately 10% [12]. The demographic, clinical, and diagnostic details, treatment, and outcome compared among the four reported patients with *S. notomytis* infection are summarized in Table 2. Fever and arthritis were the main symptoms in all cases. Our case is the first case that demonstrated meningitis, spondylodiscitis and epidural abscess as complications of *S. notomytis* infection. Our case also suggests that rat bite fever caused by *S. notomytis* presents similar clinical manifestation as those observed in rat bite fever caused by *S. moniliformis*. Moreover, rheumatoid-like polyarthritis that was initially misdiagnosed in this case has also been reported in patients with *S. moniliformis* infection [13–15]. Besides *S. notomytis*, *S. felis* is another novel species of *Streptobacillus* genus that has been reported causing rat bite fever in humans. The presentation of human *S. felis* infection from single reported case was also indistinguishable from human *S. moniliformis* infection [16].

**Table 2:** Demographic, clinical, and diagnostic details, treatment, and outcome compared among the four reported patients with *Streptobacillus notomytis* infection

	Fukushima et al. 2018 [4]	Ogawa et al. 2018 [6]	Kusuda et al. 2019 [5]	The present case 2020
Age (years)	67	94	67	72
Gender	Female	Female	Male	Female
City, country	Okinawa, Japan	Nara, Japan	Tokyo, Japan	Bangkok, Thailand
Comorbidities	Hypertension Dyslipidemia	None	Hypertension	Cervical spondylosis
Clinical manifestations	Fever Rash Polyarthritis	Fever Oligoarthritis	Fever Rash Polyarthritis	Fever Polyarthritis Meningitis Hepatitis Spondylodiscitis
Positive specimens	Blood Pus	Synovial fluid Intraoral swab from rats in the patient’s house	Blood	Synovial fluid Tissue biopsy from disc L4–L5
Identification technique	Culture and PCR	Culture and PCR	Culture and PCR	PCR
Treatment	Ampicillin 16 days	Ceftriaxone 15 days Ampicillin/sulbactam 14 days Minocycline	Meropenem 14 days	Ceftriaxone 30 days Amoxicillin 12 weeks
Outcome	Complete recovery	Died from *Acinetobacter baumannii* pneumonia	Complete recovery	Complete recovery

Similar to *S. moniliformis*, *S. notomytis* was reported to be susceptible to many classes of antibiotics [4–6]. Since culture was unable to detect the organism in our case, susceptibility testing could not be performed. However, our patient’s responsiveness to ceftriaxone indicates pathogen susceptibility. The slow response that required a longer duration of treatment is likely due to multiple sites of infection.

In summary, we have reported a case of rat bite fever caused by *S. notomytis* that was diagnosed by PCR from both synovial fluid from the right knee, and from tissue biopsy from the vertebral disc. This case demonstrates that complicated diseases, such as meningitis, spondylodiscitis, and epidural abscess can be found in *S. notomytis* infection—similar to *S. moniliformis* infection. These infections have been misdiagnosed as autoimmune disease due to manifestations that mimic rheumatoid arthritis. The diagnosis is difficult and molecular diagnosis is needed, especially in patients with no history of animal contact.

## Data Availability

The sequence of 16S rRNA was submitted to GenBank under accession number MZ676036.

## Abbreviations

CSF: Cerebrospinal fluid
PCR: Polymerase chain reaction
LS: Lumbosacral
T: Thoracic
L: Lumbar
